# Understanding child and parent perceptions of barriers influencing children’s active school travel

**DOI:** 10.1186/s12889-018-5874-y

**Published:** 2018-08-22

**Authors:** Katherine Wilson, Andrew F. Clark, Jason A. Gilliland

**Affiliations:** 1 Human Environments Analysis Laboratory, Department of Geography, University of Western Ontario, 1151 Richmond St, London, N6A 3K7 Canada; 2 Children’s Health Research Institute, 800 Commissioners Rd. E, London, N6C 2V5 Canada; 3 School of Health Studies, Department of Paediatrics, & Department of Epidemiology & Biostatistics, University of Western Ontario, 1151 Richmond St, London, N6A 3K7 Canada; 4 Lawson Health Research Institute, 750 Base Line Rd. E, London, N6C 2R5 Canada

**Keywords:** Children, Active school travel, Perceptions, Barriers, Multivariate analysis, Environments, Walking, Cycling

## Abstract

**Background:**

Physical activity plays a fundamental role in the health and well-being of children. Walking is the most common form of physical activity and the journey to and from school provides an opportunity for children to be active every day. This study examines how child and parent perceptions of barriers to active school travel influences children’s behaviour.

**Methods:**

Participants were recruited from 48 elementary schools in Southwestern Ontario, Canada. The study sample includes 1296 children (ages 9–14 years) who live within walking distance of their school, defined as 1.6 km network distance. Chi-square analysis examined differences between child and parent perceptions of barriers to active school travel. Logistic regression models examined how parent and child perceptions of barriers influence active school travel behaviour, while controlling for key intrapersonal, interpersonal, and physical environment factors.

**Results:**

The results indicate that there are significant differences in how parents and children perceive barriers to active school travel. Model results find older children, children without siblings, households with no vehicles, and children who live closer to school are most likely to use active school travel. Parent perceptions of barriers are found to have a greater influence on children’s active school travel behaviour than children’s perceptions. Different perceptions of barriers influence active school travel to school compared to returning home from school.

**Conclusions:**

Child and parent perceptions of barriers to active school travel differ and have different impacts on children’s travel behaviour. Understanding how child and parent perceptions of barriers differ can help policymakers and practitioners develop specialized interventions aimed at increasing children’s use of active school travel and children’s overall physical activity. Interventions used to promote active school travel should focus on safety, as well as perceptions of distance to break parental habits of routinely driving their children to school. Overall, this study highlights the importance of considering both child and parent perceptions to create a safe and accessible environment to allow for an increase in active school travel behaviour among elementary school children who live within walking distance of their school.

## Background

It is widely accepted that physical activity (PA) is beneficial for children’s health [1–5]. Regular PA lowers risk of chronic diseases such as obesity, coronary heart disease, Type II diabetes, and socio-psychological problems such as anxiety and depression [6–8]. Despite the known benefits of PA, few Canadian children achieve Canada’s recommended PA guidelines of at least 60 min of moderate-to-vigorous physical activity (MVPA) daily [9]. A focus on active school travel (AST) is appropriate knowing that adequate participation in PA during childhood and adolescence could be critical to the prevention of chronic disease later in life [10].

AST can be defined as any form of human-powered transportation, such as walking, biking, and skateboarding [11]. Children who actively commute to school often attain a higher daily average of MVPA [12, 13]. Furthermore, children who use AST tend to be more physically active and are more likely to meet daily recommendations of MVPA [10, 14, 15] and receive as much as 45 additional minutes of MVPA per day [14]. Despite the known benefits of AST on children’s health, AST behaviour continues to decline [16, 17]. Reversing these trends is possible if researchers can identify the barriers preventing children from walking or biking to school [18]. Previous research suggests that individual’s perceptions of their environments have a stronger and more direct relationship with children’s AST, compared to objectively measured factors [19]. Research investigating children’s independent mobility as a way to promote AST and PA has been suggested [20–22], which can be accomplished by better understanding the perceptions of barriers encountered on the journey to and from school. In the AST literature perceived barriers are defined as a person’s estimated level of challenges related to intrapersonal, interpersonal, environmental, and policy obstacles to AST [18, 23].

Studies examining children’s perceptions of their environments reveal that they possess meaningful and insightful contributions [24, 25]. Other research has shown that children are extremely cognizant of the links between health, PA, and AST [26–28]; however, few studies have examined children’s perspectives on AST [28] and fewer studies have assessed the importance and comparison among both child and parent perceptions [29–32] and the influence these perceptions have on children’s AST [33, 34].

The literature identified distance and safety as the most important barriers to AST. Children are more likely to use AST if their school is nearby and the route is safe [14, 35, 36]. Parents also play an important role in children’s AST decisions, with children whose parents use active transportation are more likely to do so [12, 37]. It has been suggested that future work should also examine the impact varying socioeconomic status (SES) and different types of urbanicity (urban, suburban, rural) have on children’s AST [10], along with a wide range of personal, family, and social factors [38, 39].

A socio-ecological approach that combines individual, interpersonal, and community factors may be the most beneficial form of research in this field [40]. AST is a complex behaviour and using a socio-ecological framework can help to explain how varying levels of features from intrapersonal, interpersonal, environmental, and policy levels influence children’s perceptions of features on their journey to and from school [41, 42]. Currently, there are inconsistencies in methodological approaches within AST research and it remains equivocal to further understand the factors influencing children’s AST [43]. It has also been suggested that future studies should examine parental perceptions and characteristics of the route to school [44]. A growing body of research conducted over the last decade, assesses the relationship between perceived barriers and rates of children’s AST. This research is vital for the development of strategies to improve the overall health and well-being of students, simply by increasing rates of PA.

This study aims to fill gaps in the current literature with the inclusion of both parent and children perceptions of barriers to AST and examining how these perceptions of barriers differ, while accounting for factors at the intrapersonal, interpersonal, and physical environment level. The two objectives of this study, include (1) to evaluate how perceptions of barriers to AST differ between children and their parents, and (2) to analyze how child and parent perceptions of barriers to AST influence how children get to school, while controlling for age, gender, distance between home and school, family composition, and neighbourhood SES.

## Methods

This study draws from two projects: Active and Safe Routes to School (ASRTS) and Spatial Temporal Environment and Activity Monitoring (STEAM) project. ASRTS is an ongoing four-year community collaborative research project within 26 schools in Southwestern Ontario, Canada examining AST of elementary school children (activesaferoutes.ca). This program aims to increase student’s AST participation through location specific targeted interventions and schools are selected based on their interest in the program. Presentations are given in every classroom in participating schools and children are sent home with a letter of information and parent survey. Older children in grades 4 to 8 are also given the opportunity to complete a child survey if they receive parental consent and provide their own assent. The final sample includes 3748 children (recruitment rate: 41.7%), with 1660 completed child surveys and 3613 completed parent surveys.

The STEAM project (steamproject.ca) is a study conducted between 2010 and 2013 within 33 schools across Southwestern Ontario examining environmental influences on children’s health and well-being. Further details of this mixed methods project are available elsewhere [45]. Schools were randomly selected from stratified groups based on neighborhood income, with 33 of the 63 schools (52.4%) that were contacted agreeing to participate. Presentations were given in grade 5 to 7 classrooms at participating schools, and letters of information, parent surveys, and parental consent forms were distributed to students. All students with consent were asked to provide assent and complete the child survey. The final sample includes 932 children (recruitment rate 66.9%), with 876 completed child surveys and 758 completed parent surveys.

Data provided by these two studies are collected from parent and child surveys that ask identical questions about AST. The parent survey includes questions about family SES, postal code, the journey to and from school, and perceptions of barriers to AST. The child survey includes questions about child and family demographics and perceptions of barriers to AST. These surveys includes previously-validated questions assessing AST and children’s perceptions of their local environments from other widely-used surveys, including Neighborhood Quality of Life Study and the Neighbourhood Environment Walkability Scale [46–49].

### Sample

The sample used in this study will combine data from the baseline survey of the two projects (*N* = 4680) from 48 elementary schools across Southwestern Ontario, Canada (i.e., City of London, Middlesex County, City of St. Thomas, Elgin County, Oxford County, Chatham-Kent, City of Sarnia, Lambton County, and Essex County). There are two exclusion criteria for this study. Observations without a parent and child survey completed were removed from the sample, resulting in 2501 children removed from the sample. All children who are bus eligible, defined as living more than 1.6 km from school [44], were excluded from the sample, resulting in 883 more children removed from the sample. Applying the exclusion criteria results in a final sample of 1296 paired parent and child who live within 1.6 km of their school. The characteristics of the final sample can be found in Table 1.

### Measures

#### Dependent variable

There is variation within the literature on consistency of definitions and measurements of AST used in research. A systematic review conducted by Lu and collegues [18] on AST states that although there is currently no “golden rule” for defining AST, it is necessary for researchers to provide a valid rationale for how AST is defined in their research. The dependent variable for this analysis is parent-reported rates of AST from the parent surveys, with students classified as an active traveler if they identified their mode of travel as active more than 50% of their total trips. A 50% cut-off is used for this study, as the results are being used in the development of an intervention that has the goal of getting every child who lives within walking distance to walk on a regular basis. Modes considered active for the purposes of this study include walking, bicycling, scootering, skateboarding, and rollerblading. Inactive modes included car or personal vehicle, with children who take a bus being excluded from this study. From this definition of AST, two dependent variables were developed: (a) AST to school more than 50% of the total trips; (b) AST from school for more than 50% of the total trips.

#### Independent variables

Following the socio-ecological framework, this study uses three levels of independent variables that will be used as control variables: intrapersonal, interpersonal, and physical environment [41, 50]. Policy is controlled for by excluding all children who live outside of the walk zone of each school.

**Intrapersonal** variables were collected on both the parent and child surveys. Answers primarily come from the child surveys, with answers from the parent surveys being used when responses were missing. The variables used in this study are measured for each child: **gender** (i.e., child self-identifying as a *girl* [0] or *boy* [1]); and **age** (i.e., continuous measure of age in years).

**Interpersonal** variables were collected from parent or child surveys and measured for each child:

- **Parent Education:** Parents (mother and father) self-identified *no high school diploma* (0), *high school diploma* (1), or *post-secondary education* (2). Completing any education past high school is considered post-secondary education.
- **Employment Status:** Parents (mother and father) self-identified as being *unemployed* (0) or *employed* (1). Being employed includes employed full-time, employed part-time, while unemployed includes at home with children, unemployed, or student.
- **Siblings:** Child self-identified the number of children living in their household, with one child represents *no siblings* (0) and many children represents *siblings* (1).
- **Number of Vehicles:** parent self-identified the number of motor vehicles in their household, including *not owning a vehicle* (0), *owning one vehicle* (1), and *owning 2 or more vehicles* (2).
- **Median Family Income:** Neighbourhood level median family income from the 2011 National Household Survey [51], measured by the census dissemination area in which a child’s home is located (i.e., home postal code).

The **Physical Environment** are measured in two ways:

- **Distance from home to school:** the network distance in kilometres between home (i.e., home postal code) and school (i.e., point at centre of building footprint) were measured using the Network Analyst tool in ArcGIS 10.3 [52].
- **Level of urbanicity:** Urbanicity refers to the urban form in which each child’s home is located, including *suburban* (0), *urban* (1), and *rural* (2). Urban is defined as the City of London boundary in 1961 (i.e., year suburbanization started). Suburban is defined as the remaining area within London. Rural includes all small towns and townships with a population under 100,000 (including agricultural land).

The rest of the independent variables used in this study are parent and child **perceptions of barriers to AST**. The questions, shown in Table 2, ask respondents to specify their level of agreement or disagreement on a four-point Likert-scale and responses were converted into binary variables for analysis: *completely no and mostly no* (0) and *mostly yes and completely yes* (1). Barriers are categorized into 4 themes: physical environment, safety, social, and individual/family preferences.

**Table 1:** Descriptive Statistics of the study sample (*n* = 1296)

Variable	n	%
Active to School	914	70.5
Active from School	984	75.9
Intrapersonal		
Gender		
Girl	716	55.2
Boy	580	44.8
Age		
8	20	1.5
9	210	16.2
10	263	20.3
11	343	26.5
12	285	22.0
13	158	12.2
14	17	1.3
Interpersonal		
Mother Education Level		
No High School Diploma	48	3.7
High School Diploma	229	17.7
Post Secondary Graduate	988	76.2
Father Education Level		
No High School Diploma	61	4.7
High School Diploma	236	18.2
Post Secondary Graduate	908	70.1
Mother Occupation Status		
Unemployed	295	22.8
Employed	943	72.8
Father Occupation Status		
Unemployed	109	8.4
Employed	1072	82.7
Siblings		
No Siblings	211	16.3
Siblings	1074	82.9
Number of Vehicles in Family		
0	59	4.6
1	408	31.5
2 or more	799	61.7
Median family income in CAD (in thousands), Mean (SD)	–	79.51 (27.36)
Physical Environment		
Distance home to school (km), Mean (SD)	–	0.90 (0.395)
Urbanicity		
Suburban	753	58.1
Urban	125	9.6
Rural	418	32.3

*Note: numbers may not add to full sample size due to missing values*

**Table 2:** Perceived barriers to AST from child survey

Physical Environment	
It is too far or takes too much time	
There are not enough sidewalks	
There are not enough bike paths/ lanes	
There is nowhere to safely leave a bike if I ride my bike to school	
There are not a lot of trees along the streets in my neighbourhood	
Safety	
It feels unsafe due to traffic on the route	
Most drivers go too fast while driving in our neighbourhood	
There are too many busy streets to cross	
There is a lot of crime in our neighbourhood	
It feels unsafe to walk by myself around my neighbourhood during the day	
It feels unsafe to walk with friends or siblings in my neighbourhood during the day	
Social	
There is no one to walk or bike with	
I do not know a lot of people in my neighbourhood	
I might get bullied/ teased along the way	
Individual/ Family Preferences	
The route is too boring	
I get too hot and sweaty	
It is not fun	
I have too much stuff to carry	
It is more difficult to walk than drive	

*Questions have been reverse coded to be presented as barriers instead of facilitators*

**Table 3:** Chi-square test for independence, parent and child perception of barriers

Barriers to Active Transportation	X^2^ (p)	Both Agree n (%)	Both Disagree n (%)	Parent Agree & Child Disagree n (%)	Child Agree & Parent Disagree n (%)
Physical Environment					
Too Far / Takes too much Time	98.821 (< 0.001)	50 (4.1%)	998 (81.5%)	113 (9.2%)	64 (5.2%)
Not enough sidewalks	74.701 (< 0.001)	71 (6.3%)	819 (73.2%)	127 (11.3%)	102 (9.1%)
Not enough bike paths	32.325 (< 0.001)	134 (11.3%)	631 (53.2%)	290 (24.5%)	130 (11.0%)
No bike rack	11.954 (< 0.001)	44 (3.7%)	868 (72.8%)	165 (13.8%)	116 (9.7%)
Not a lot of trees	26.767 (< 0.001)	119 (9.7%)	700 (57.0%)	161 (13.1%)	248 (20.2%)
Safety					
Route feels unsafe due to traffic	82.642 (< 0.001)	145 (12.0%)	662 (54.8%)	330 (27.3%)	72 (6.0%)
Drivers speed on streets	54.774 (< 0.001)	289 (23.4%)	374 (30.2%)	500 (40.4%)	74 (6.0%)
Too many busy streets	77.415 (< 0.001)	94 (7.8%)	806 (67.0%)	221 (18.4%)	82 (6.8%)
Feels unsafe because of crime	39.975 (< 0.001)	33 (2.7%)	994 (81.7%)	127 (10.4%)	62 (5.1%)
Unsafe for child to walk alone	27.063 (< 0.001)	64 (5.2%)	827 (67.4%)	255 (20.8%)	81 (6.6%)
Unsafe for child to walk with friends	84.162 (< 0.001)	71 (5.8%)	911 (74.5%)	86 (7.0%)	154 (12.6%)
Social					
No one to walk with	21.698 (< 0.001)	57 (4.8%)	851 (71.0%)	157 (13.1%)	133 (11.1%)
I do not know people in my neighbourhood	53.527 (< 0.001)	141 (11.4%)	709 (57.3%)	224 (18.1%)	164 (13.2%)
Might get bullied/teased	46.066 (< 0.001)	46 (3.8%)	920 (76.2%)	184 (15.2%)	57 (4.7%)
Individual/ Family Preferences					
Route is boring	15.367 (< 0.001)	27 (2.3%)	945 (78.8%)	56 (4.7%)	171 (14.3%)
Get too hot/sweaty	21.137 (< 0.001)	28 (2.3%)	975 (81.3%)	73 (6.1%)	124 (10.3%)
Not fun to walk	24.158 (< 0.001)	28 (2.3%)	973 (81.1%)	59 (4.9%)	140 (11.7%)
Too much stuff to carry	29.217 (< 0.001)	66 (5.5%)	834 (69.4%)	186 (15.5%)	116 (9.7%)
More difficult to walk than drive	134.404 (< 0.001)	198 (16.4%)	669 (55.4%)	163 (13.5%)	177 (14.7%)

**Table 4:** Logistic regression analysis to understand the impact intrapersonal, interpersonal, and physical environmental factors have on AST behaviour

	Active Travel to School			Active Travel from School		
	OR	95% CI	*P*	OR	95% CI	*P*
Intrapersonal						
Boys (ref: girls)	1.234	0.919–1.655	0.162	1.146	0.902–1.455	0.264
Age	1.394	1.254–1.549	*< 0.001*	1.486	1.344–1.643	*< 0.001*
Interpersonal						
Mothers Education (ref: no high school diploma)						
High school diploma	1.106	0.489–2.502	0.810	1.245	0.464–3.338	0.664
Post secondary	1.414	0.645–3.100	0.387	1.096	0.462–2.601	0.835
Missing	2.478	0.708–8.672	0.156	1.575	0.296–8.392	0.594
Fathers Education (ref: no high school diploma)						
High school diploma	0.600	0.269–1.336	0.211	1.003	0.520–1.933	0.993
Post secondary	0.900	0.405–2.000	0.797	1.648	0.844–3.219	0.144
Missing	0.912	0.330–2.520	0.859	1.225	0.395–3.798	0.726
Siblings (ref: No Siblings)	0.988	0.979–0.997	*0.007*	0.988	0.977–0.999	*0.039*
Mothers Occupational Status (ref: Unemployed)						
Employed	1.410	1.030–1.929	*0.032*	1.490	1.052–2.111	*0.025*
Missing/prefer not to answer	1.082	0.552–2.121	0.818	1.221	0.602–2.477	0.579
Fathers Occupational Status (ref: Unemployed)						
Employed	1.412	0.862–2.313	0.170	1.417	0.757–2.651	0.276
Missing/prefer not to answer	1.151	0.543–2.440	0.714	1.369	0.606–3.094	0.450
Number of Vehicles (ref: 0 vehicles)						
1	0.175	0.062–0.490	*< 0.001*	0.211	0.072–0.618	*0.005*
2 or more	0.105	0.037–0.295	*< 0.001*	0.134	0.045–0.397	*< 0.001*
Missing	0.283	0.076–1.062	0.061	0.322	0.087–1.194	0.090
Median Family Income (10,000 CAD)	0.951	0.888–1.019	0.155	0.949	0.873–1.032	0.221
Physical Environment						
Network distance between home and school (km)	0.163	0.096–0.277	*< 0.001*	0.167	0.094–0.298	*< 0.001*
Urbanicity (ref: Suburban Large City)						
Urban	0.589	0.348–0.996	*0.048*	0.851	0.400–1.809	0.675
Rural	0.845	0.495–1.443	0.538	0.804	0.454–1.424	0.454
Constant	2.207	0.374–13.012	0.382	0.896	0.129–6.226	0.912
Sample size (N)	1292			1273		
Pseudo R2	0.135			0.142		
Log Pseudolikelihood	− 676.545			−586.435		

*All italicized numbers represent statistical signifigance based on critical value cut-offs p<0.05 or p<0.01*

**Table 5:** Univariate logistic regression analysis to understand the impact perceived barriers have on AST behaviour while controlling for intrapersonal, interpersonal, and physical environmental factors

	Active Travel to School						Active Travel from School					
	Parent Perceptions			Child Perceptions			Parent Perceptions			Child Perceptions		
	OR	95% CI	*P*	OR	95% CI	*P*	OR	95% CI	*P*	OR	95% CI	*P*
Physical Environment												
Too Far / Takes too much Time	0.197	0.125–0.312	*< 0.001*	0.690	0.401–1.186	0.179	0.296	0.197–0.446	*< 0.001*	0.470	0.283–0.781	*0.004*
Not enough sidewalks	0.773	0.587–1.017	0.066	0.802	0.540–1.190	0.272	0.824	0.574–1.183	0.294	0.648	0.419–1.000	*0.049*
Not enough bike paths	0.637	0.501–0.809	*< 0.001*	0.685	0.478–0.982	*0.040*	0.689	0.508–0.936	*0.017*	0.610	0.403–0.923	*0.019*
No bike rack	0.709	0.456–1.102	0.126	0.806	0.575–1.132	0.214	0.907	0.586–1.403	0.660	0.710	0.529–0.952	*0.022*
Not lots of trees	0.963	0.692–1.341	0.825	0.778	0.615–0.985	*0.037*	1.079	0.788–1.477	0.637	0.761	0.535–1.082	0.129
Safety												
Route feels unsafe due to traffic	0.306	0.233–0.402	*< 0.001*	0.608	0.440–0.841	*0.003*	0.343	0.260–0.451	*< 0.001*	0.535	0.358–0.801	*0.002*
Drivers speed on streets	0.751	0.598–0.943	*0.014*	0.894	0.716–1.116	0.322	0.798	0.606–1.050	0.108	0.706	0.504–0.990	*0.043*
Too many busy streets	0.352	0.254–0.487	*< 0.001*	0.585	0.399–0.859	*0.006*	0.314	0.222–0.446	*< 0.001*	0.501	0.373–0.673	*< 0.001*
Feels unsafe because of crime	0.490	0.300–0.803	*0.005*	0.651	0.422–1.006	0.053	0.368	0.229–0.593	*< 0.001*	0.439	0.259–0.745	*0.002*
Unsafe for child to walk alone	0.372	0.274–0.504	*< 0.001*	0.498	0.354–0.699	*< 0.001*	0.398	0.303–0.524	*< 0.001*	0.482	0.330–0.705	*< 0.001*
Unsafe for child to walk with friends	0.464	0.302–0.713	*< 0.001*	0.727	0.429–1.233	0.237	0.380	0.274–0.526	*< 0.001*	0.604	0.381–0.955	*0.031*
Social												
No one to walk with	0.244	0.190–0.315	*< 0.001*	0.535	0.362–0.791	*0.002*	0.264	0.187–0.372	*< 0.001*	0.462	0.309–0.690	*< 0.001*
I do not know people in my neighbourhood	0.723	0.534–0.980	*0.036*	0.813	0.607–1.088	0.164	0.625	0.444–0.881	*0.007*	0.679	0.477–0.966	*0.032*
Might get bullied/ teased	0.448	0.316–0.634	*< 0.001*	0.570	0.372–0.874	*0.010*	0.418	0.283–0.616	*< 0.001*	0.541	0.336–0.868	*0.011*
Individual/ Family Preferences												
Route is boring	1.080	0.664–1.757	0.757	0.896	0.632–1.270	0.536	1.022	0.619–1.690	0.931	0.799	0.515–1.239	0.316
Get too hot/sweaty	0.708	0.319–1.570	0.395	0.831	0.596–1.159	0.276	0.723	0.409–1.278	0.264	0.620	0.432–0.888	*0.009*
Not fun to walk	0.602	0.318–1.140	0.119	0.746	0.516–1.077	0.118	0.643	0.421–0.981	*0.041*	0.479	0.310–0.741	*0.001*
Too much stuff to carry	0.362	0.265–0.494	*< 0.001*	0.664	0.432–1.020	0.061	0.312	0.222–0.439	*< 0.001*	0.541	0.350–0.834	*0.005*
More difficult to walk than drive	0.099	0.065–0.150	*< 0.001*	0.191	0.141–0.257	*< 0.001*	0.146	0.100–0.213	*< 0.001*	0.225	0.156–0.326	*< 0.001*

*All italicized numbers represent statistical signifigance based on critical value cut-offs p<0.05 or p<0.01*

**Table 6:** Multivariate logistic regression analysis to understand the impact perceived barriers have on AST behaviour while controlling for intrapersonal, interpersonal, and physical environmental factors

Variable	Active Travel to School						Active Travel from School					
	Parent Perceptions			Child Perceptions			Parent Perceptions			Child Perceptions		
	OR	95% CI	P	OR	95% CI	*P*	OR	95% CI	*P*	OR	95% CI	*P*
Barriers: Physical Environment												
Too far / Takes too much time	0.399	0.216–0.738	*0.003*	–	–	–	0.577	0.322–1.034	0.064	0.768	0.487–1.211	0.256
Not enough bike paths	1.159	0.771–1.742	0.479	0.942	0.606–1.465	0.791	1.312	0.861–1.999	0.207	1.015	0.622–1.657	0.951
Not enough sidewalks	–	–	–	–	–	–	–	–	–	0.861	0.577–1.285	0.464
No bike rack	–	–	–	–	–	–	–	–	–	1.168	0.761–1.792	0.477
Not lots of trees	–	–	–	0.766	0.589–0.998	*0.048*	–	–	–	–	–	–
Barriers: Safety												
Route feels unsafe due to traffic	0.590	0.345–1.008	0.053	1.034	0.640–1.671	0.892	0.906	0.550–1.490	0.697	0.905	0.476–1.719	0.760
Too many busy streets	0.779	0.470–1.291	0.333	1.134	0.625–2.059	0.678	0.589	0.344–1.010	0.054	1.042	0.533–1.966	0.898
Unsafe for child to walk alone	0.695	0.448–1.080	0.106	0.627	0.392–1.005	0.052	0.991	0.652–1.507	0.968	0.857	0.562–1.307	0.473
Unsafe for child to walk with friends	1.217	0.616–2.405	0.572	–	–	–	0.710	0.434–1.161	0.173	0.852	0.426–1.704	0.651
Drivers speed on streets	1.415	1.032–1.940	*0.031*	–	–	–	–	–	–	1.326	0.942–1.865	0.105
Feels unsafe because of crime	0.837	0.393–1.782	0.644	–	–	–	0.638	0.335–1.214	0.171	0.446	0.233–0.851	*0.014*
Barriers: Social												
No one to walk with	0.523	0.355–0.768	*< 0.001*	0.677	0.443–1.034	0.071	0.517	0.300–0.894	*0.018*	0.621	0.374–1.032	0.066
Might get bullied/teased	1.133	0.719–1.784	0.592	0.902	0.559–1.456	0.672	1.056	0.626–1.784	0.836	0.926	0.579–1.482	0.749
I do not know people in my neighbourhood	1.162	0.829–1.630	0.383	–	–	–	1.022	0.695–1.503	0.910	0.906	0.617–1.330	0.614
Barriers: Individual/ Family Preferences												
More difficult to walk than drive	0.130	0.083–0.205	*< 0.001*	0.197	0.141–0.276	*< 0.001*	0.197	0.123–0.315	*< 0.001*	0.243	0.168–0.352	*< 0.001*
Too much stuff to carry	0.936	0.558–1.568	0.801	–	–	–	0.551	0.343–0.886	*0.014*	0.758	0.451–1.273	0.294
Get too hot/ sweaty	–	–	–	–	–	–	–	–	–	1.276	0.844–1.930	0.248
Not fun to walk	–	–	–	–	–	–	2.449	1.252–4.792	*0.009*	0.917	0.575–1.461	0.715
Constant	118.737	7.562–1864.471	0.001	4.066	0.385–42.976	0.244	41.369	1.537–832.813	0.020	2.100	0.124–35.486	0.607
Sample size (N)	1169			1188			1172			1046		
Pseudo R2	0.333			0.233			0.297			0.255		
Log Pseudolikelihood	−470.972			− 547.689			−439.301			−419.026		

*All italicized numbers represent statistical signifigance based on critical value cut-offs p<0.05 or p<0.01*

### Statistical analysis

Two analyses were completed as part of this study to achieve the study objectives: chi-square tests and logistic regression. To achieve objective 1, chi-square tests for independence were completed in IBM SPSS Statistics 24 [53] to explore the relationship between parent and child responses to questions about perceptions of barriers. A chi-square test is applied when there are two categorical variables from a single population (parent and child perception of individual barriers) and is used to determine if there is a significant association between the two variables.

To achieve objective 2, a series of logistic regression models were computed in STATA SE 13 64bit [54]. Logistic regression has been chosen as it is more robust and does not have the assumptions (e.g., normal distribution, equal variance) many other binary type analyses include (e.g., logit, probit) [55]. Further, logistic regression parameter estimates can be converted into odds ratios, which are interpreted as the odds of success of the outcome variable over its failure [56]. All logistic regression models in this study included estimates for robust standard errors to account for clustering at the school level [57]. Logistic regression models were computed for both (a) to school AST and (b) from school AST using the following step-wise process: (1) Intrapersonal, interpersonal, and physical environment; (2) Model 1 + univariate barriers; (3) Model 2 + All significant univariate barriers for parents; (4) Model 3 + all significant univariate barriers for children. Statistical significance is reported based on critical value cut-offs of 0.05 and 0.01.

## Results

### Chi-Square analysis

The chi-square analysis comparing parent and child perceptions found a statistically significant difference in the distribution of every barrier (Table 3). This shows that parents and children have varying perceptions of barriers influencing a child’s journey to and from school, although it should be noted that there is still a high percentage of matching agreement or disagreement between parents and children (i.e., average of 77.3% of parents and children have matching agreement/disagreement). Trends of these varying perceptions can be seen within the four barrier themes, including physical environment, safety, social, and individual/family preferences.

Within the theme of physical environment perceptions of the barriers **not enough bike paths** and **not enough trees** on children’s routes to school have the largest differences of responses between parent and child*.* Most parents and children believe there are enough bike racks at the school and do not perceive this to be a barrier to AST. The remaining three themes saw larger discrepancies between child and parent perceptions of barriers. Parents perceived all safety related questions as barriers to their children’s AST, while children did not. The exception being the barrier **unsafe for a child to walk with a friend**, where parents and children held opposite responses compared to all other safety related barriers. Within the theme of social barriers, the largest variation of responses between parent and child was seen in **bullying**. Children did not see being bullied or teased as a barrier, while parents did. Finally, within the theme of individual/ family preferences children perceived more of these as barriers to AST than their parents. The exception being **too much stuff to carry,** with parents perceiving this as more of a barrier than children. These results demonstrate significant differences between parent and child perceptions of barriers, although, it is still unknown whether these perceived barriers influence the decision of children to use AST.

### Intrapersonal, interpersonal, and physical environment factors

Following the social-ecological framework, a logistic regression model was conducted to understand how the intrapersonal, interpersonal, and physical environment factors influence AST for this sample (Table 4). Across all levels of the social-ecological framework, there is little difference between significant factors that influence the journey to school and from school. Age was found to be significantly related to an increase in the chances of children using AST. Multiple interpersonal factors found significantly related to a decrease in the odds of using AST both to and from school, including having siblings and more vehicles, while a mother being employed significantly increases the odds of using AST. Distance is related to AST both to and from school, with children living farther away decreasing the odds of choosing AST. The only variable that has different results between to and from school is urbanicity, where children living in urban areas have significantly lower odds of using AST when traveling to school than children living in suburban areas.

### Univariate models

The univariate logistic regression models estimate the relationship between AST to and from school and each of the parent and child perceptions of the 19 barriers independently, while controlling for factors at the intrapersonal, interpersonal, and physical environment (Table 5). On the journey to school, parent’s perceptions of barriers explained AST more than children’s perceptions of barriers, particularly when examining safety and social influences. Parent’s perceptions of all safety and social barriers included in this univariate model are found to significantly impact children’s AST. Perceptions of barriers on the journey home from school showed opposite outcomes, with children’s perceptions of barriers explaining AST more than parent’s perceptions of barriers. Children’s perceptions of all barriers included in the univariate model except **not enough trees** and **route is boring** showed statistical significance. Similar to parent’s perceptions, results show that children’s perceiving barriers are less likely to use AST. Perceptions of safety have the greatest impact on AST behaviour to and from school for both children and parents.

### Multivariate models

All barriers statistically significant in the univariate analysis were included in the final multivariate models, while controlling for intrapersonal, interpersonal, and physical environment factors. Results for the four final models are shown in Table 6, including AST to and from school for both parent and child perceptions.

There is variation between parent and child perceptions of barriers on the **journey to school** and the impact they have on AST. From the physical environment barriers parents perceive **too far/ takes too much time** as a barrier that negatively impacts children’s use of AST on their journey to school. Children perceive the physical environment barrier **there are not a lot of trees along the streets in my neighbourhood** to have a negative impact on their AST behaviours. Parent’s perceptions of safety barriers are explaining AST more than children’s perceptions of safety barriers on the journey to school. The safety barrier **drivers speeding on the streets** positively impacts children’s use of AST. From the social barriers, parents perceive the barrier of their children having **no one to walk with** to negatively impact children’s AST. Finally, both parents and children see the individual/ preference barrier **more difficult to walk than drive** as having a negative impact on AST a preference relating to their journey to school. In general, parent’s perceptions of barriers on the journey to school explain AST behaviours more than children’s perceptions of barriers.

Within this final model, parents and children did not perceive any physical environment barriers to impact children’s AST on the **journey home from school**. **It feels unsafe because of crime** is a safety barrier that children believe to negatively impact use of AST. From the social barriers, similar to the journey to school, parents perceive the barrier **no one to walk with** to negatively impact children’s AST. Finally, from the individual/ family preference barriers, parents perceive **not fun to walk** and **too much stuff to carry** to impact children’s use of AST on the journey home from school. As well, both parents and children see the barrier, **more difficult to walk than drive** to have a negative impact on AST. Parents and children’s perceptions of barriers share similarities between the journey to and from school however, more barriers are perceived to have an impact on AST on the journey home from school.

## Discussion

This study compared parent and child perceptions of barriers and how they influenced children’s AST, while controlling for intrapersonal, interpersonal, and physical environment variables. Age, siblings, mothers occupational status, vehicle ownership, distance between home and school, and urbanicity were control variables found to impact children’s use of AST. In general, parents perceive more barriers to AST than children, although both parents and children perceptions have an impact on AST behaviour. There are also differences in the impact barriers have between the journey to school and home from school.

The intrapersonal, interpersonal, and physical environment variables are mostly consistent with previous research. At the intrapersonal level, age is found related to an increase in AST behaviour on the journey to and from school [58–61] and there is no significant difference between boys and girls [10, 62]. There are multiple variables significant at the interpersonal level, as children are known to be largely influenced by the context of their family [63]. Children’s independent mobility, including AST, is associated with having siblings in both previous research and this study [64]. When children have employed mothers they have higher rates of AST, as children rely heavily on their mothers for transportation [11]. Having no car in the household is also related to an increase in AST, as these children really have few other options [11]. Interventions need to focus on getting children out of the car and into active modes, as congestion and vehicle emissions around the schools are the primary concerns for planning and transportation decisions [65].

There are also physical environment factors significantly related to AST, including distance and urbanicity. As in past research, distance between home and school is the strongest predictor of children’s AST [11, 39, 44, 66, 67], even when only examining those who live within walking distance of the school. Urbanicity is also found to significantly influence AST on the way to school, with children living in urban environments significantly less likely to use AST on the way to school compared to children living in suburban environments.

Findings show us that there is variation between parent and child perceptions of barriers on the journey to and from school and the impact they have on AST. The univariate analysis finds that parents perceptions of barriers explain AST more than children’s perceptions on the journey to school, while children’s perceptions of barriers are explaining AST more than parent’s perceptions for the journey home from school. A simple explanation of this finding can be that parents influence children’s travel modes in order to coordinate with parent’s work schedules [68]. Often parents are more involved with their child’s journey to school before or on their way to work, with dropping their child off at school as a part of their own getting to work route [69]. Children’s barriers are then more likely to impact behaviour on the journey home from school when its not part of a trip chain.

Physical environment barriers were found significant for the journey to school. Parents perceive the barrier **too far/ takes too much time** as significantly influential on decreasing children’s use of AST on the journey to school. Many parents go to work in the morning, so it is reasonable to assume that if they perceive their children’s journey as time consuming or too long, they see the time savings of driving a benefit compared to the extra time it takes to walk [68]. Children perceived **not having enough trees** as a physical environment barrier on the journey to school that negatively impacts their AST. Although previous finding in the literature about tree densities have mixed findings, the majority of research suggests that trees are positively associate with an increase in walking and that the presence of street trees are positively associated with children’s AST [8, 19, 44].

Barriers relating to safety had the most significant variables during the univariate analysis, but few have significant influence on children’s AST. The only safety barrier parent’s perceived as significantly influencing children’s journey to school was **drivers speed on streets**, which positively influence children’s use of AST. Drivers speeding related to an increase in AST is opposite to what was expected [70], meaning future research should examine the reason for this reverse perception. Although, past research examining barriers to active transportation has found those being active also perceive more safety barriers [71].

Children’s perceptions of social barriers to AST were not related to the journey to or from school; social barriers were only significant when perceived by parents. Parents perceive their child having **no one to walk with** as a barrier to AST on both the journey to and from school. Zhu and Lee [72] state that supportive peer influences have been found to increase children’s AST. Parents may feel uncomfortable allowing their child to walk alone; therefore, if they do not believe there is anyone for their child to walk with (e.g., friends, peers) they might not allow their child to walk to school. Multiple studies have found that girls who reported having friends in their neighbourhood were more likely to use AST [73, 74]. Although this study classifies no one to walk with as a social barrier, parents may perceive this as a way to mitigate safety barriers.

Individual/ Family Preferences is the last theme of barriers examined in this research. Parents perceive the barrier **too much stuff to carry** to negatively influence children’s use of AST on the journey home from school. This finding is intuitive in that children have the addition of homework, as well as other things collected throughout the school day to carry home. Parents should be aware of backpack recommendations and encourage their children to wear straps on both shoulders to avoid long-term risks associated with back pain [75]. Finally, findings were consistent for both parent and child perceptions that **more difficult to walk than drive** negatively impacts children’s use of AST to and from school. This is an important perception to target when designing interventions aiming to increase rates of AST. Children who do not use AST when they are able (i.e., live within walking distance) are missing out on the many benefits that come with this action. The PA that children are able to achieve while using AST can provide them with numerous physical, psychological, emotional, and behavioral health benefits [76].

### Policy and practice

Findings from this study can be used to make recommendations for policy makers and practitioners. This research supports previous literature with results demonstrating that distance from home to school significantly influences children’s AST [77], even for student’s living within walking distance (1.6 km). This reiterates the importance of school siting decisions made by school boards, city planners, and developers [17, 44]. Shorter distance is a key influence on the mode children use to travel to and from school and should be considered by all those who have an input in deciding school locations and boundaries.

This research expands on the literature acknowledging the role children play in making decisions related to AST. Children and their parents often have varying perceptions of safety and their environments [33, 78]. Children’s positive perceptions are often associated with an increase in PA [79], and this research demonstrates the same with respect to perceptions of barriers and AST. How children’s perceive features they experience on their journey to and from school affects their decisions to use AST [78].

Successful interventions targeting levels of AST should consider influencers from all levels of the socio-ecological framework [80]. Research with a sole focus on the built environment as an intervention strategy will be insufficient in increasing AST since there is a wide range of factors that influence children’s decisions about AST. Integrating AST as a part of children’s daily routines is likely the most efficient intervention to raise children’s level of PA. Along with the involvement of children and parents, interventions should engage community partners across multiple stakeholder levels, as suggested within the socio-ecological framework [42]. The use of school travel planning (STP) is a preeminent intervention being used to promote children’s AST through collaborative public health strategies [16, 81–83]. STP is a location specific, multi-sector intervention linking together key stakeholders with school communities to create safe environments in which more children can engage in AST [84]. Findings from this research provide key barriers that STP interventions should consider to increase the successfulness of the intervention. This research demonstrates that neighbourhood environments and the perceptions parents and children have of them, matter within the context of AST. Healthy, pedestrian friendly environments and how they are perceived are an important part of supporting and increasing children’s AST [85, 86]. Our study contributes to the growing body of literature on how perceptions of barriers within local environments can influence children’s AST.

### Limitations and future work

One limitation of this study is AST is measured using data from a self-reported survey, which needs to be taken at face value despite potential biases (e.g., selective memory or exaggeration) [87, 88]. While objective measures, such as GPS tracking, would be preferred over self-report, the sample size of our study makes surveys a much more feasible way to collect AST outcome data. Another limitation of this study is the use of postal codes instead of exact home locations. This may cause slight variation within the estimations of the distance children travel from their home to school; however, previous studies indicate that postal codes are a reasonable proxy for home address in this region [89]. In the future, studies should use actual home locations, as well as GPS tracking to evaluate findings based on the actual routes children travel to and from school. Another limitation of this study is the use of self-reported measures of AST and inability to verify response accuracy. The inclusion of matched parent and child surveys allows for some validity checks, however a supplementary objective measure to avoid bias would be beneficial.

Future research should move beyond barriers and include perceptions of enablers experienced by children during AST to understand more features and the influence they may have on children’s journeys to and from school. As well, further research on perceptions of barriers to children’s AST needs to be completed using qualitative research methods. Although this study provides us with a deeper understanding of both parent and child perceptions of AST, a key finding is that that there is a difference between these perceptions. The decision to use AST is a complex one and can be influenced by multiple factors. It is possible that children may have other perceived barriers not asked on the survey, thus providing justification for the need of individualized qualitative research to design effective interventions at schools.

## Conclusions

This study makes multiple contributions to the literature on children’s AST. We found that a combination of perceptions on environmental, safety, social, and preference barriers influence children AST. Our findings suggest that interventions used to promote AST should focus on safety, as well as perceptions of distance to break through habits of routinely getting driven to school. Interventions aiming to increase AST should include both parent and children in the process. Results from this study highlight that the relationships between PA levels and transport modes may vary within and across different populations and that AST initiatives should be tailored accordingly [10, 90]. This study contributes to the growing body of research of how local environments can influence children’s AST. Results show the importance in acquiring children’s perspectives when researching a topic involving them, as varying opinions can be seen between parents and their children. The importance of children’s perceptions and opinions should be valued because creating a safe and accessible environment for children is creating a healthy environment for all.

## Abbreviations

ASRTS: Active and Safe Routes to School
AST: Active School Travel
MVPA: Moderate to Vigorous Physical Activity
PA: Physical Activity
SES: Socioeconomic Status
STEAM: Spatial Temporal Environment and Activity Monitoring
STP: School Travel Planning

## References

[1] de Moraes Ferrari GL, Oliveira LC, Araujo TL, Matsudo V, Barreira TV, Tudor-Locke C (2015). Moderate-to-vigorous physical activity and sedentary behavior: independent associations with body composition variables in Brazilian children. Pediatr Exerc Sci.

[2] Herman KM, Chaput J-P, Sabiston CM, Mathieu M-E, Tremblay A, Paradis G (2015). Combined physical activity/sedentary behavior associations with indices of adiposity in 8- to 10-year-old children. J Phys Act Health.

[3] Biddle SJH, Asare M. Physical activity and mental health in children and adolescents: a review of reviews. Br J Sports Med. 2011;45:886-95. 10.1136/bjsports-2011-090185 21807669

[4] Larun L, Nordheim LV, Ekeland E, Hagen KB, Heian F. Exercise in prevention and treatment of anxiety and depression among children and young people. Cochrane Database Syst Rev. 2006; Available from: http://doi.wiley.com/10.1002/14651858.CD004691.pub2 10.1002/14651858.CD004691.pub2 PMC12742371 16856055

[5] McIsaac J-L, Kirk S, Kuhle S (2015). The association between health Behaviours and academic performance in Canadian elementary school students: a cross-sectional study. Int J Environ Res Public Health.

[6] Figueroa-Muñoz JI, Chinn S, Rona RJ (2001). Association between obesity and asthma in 4–11 year old children in the UK. Thorax.

[7] Janssen I, Leblanc AG. Systematic review of the health benefits of physical activity and fitness in school-aged children and youth. Int J Behav Nutr Phys Act. 2010;7. Available from: https://www.ncbi.nlm.nih.gov/pmc/articles/PMC2885312/. 10.1186/1479-5868-7-40 PMC2885312 20459784

[8] Larsen K, Gilliland J, Hess PM (2012). Route-based analysis to capture the environmental influences on a Child’s mode of travel between home and school. Ann Assoc Am Geogr.

[9] Tremblay MS, Warburton DE, Janssen I, Paterson DH, Latimer AE, Rhodes RE (2011). New Canadian physical activity guidelines. Appl Physiol Nutr Metab NRC Res Press Appl Physiol Nutr Metab.

[10] Faulkner GEJ, Buliung RN, Flora PK, Fusco C (2009). Active school transport, physical activity levels and body weight of children and youth: a systematic review. Prev Med.

[11] McDonald NC (2007). Active transportation to school. Trends among U.S. schoolchildren, 1969-2001. Am J Prev Med.

[12] Henne HM, Tandon PS, Frank LD, Saelens BE. Parental factors in children’s active transport to school. Public Health. 2014;128:643-6. 10.1016/j.puhe.2014.05.004 PMC4149318 24999161

[13] Mendoza JA, Watson K, Nguyen N, Cerin E, Baranowski T, Nicklas TA (2011). Active commuting to school and association with physical activity and adiposity among US youth. J Phys Act Health.

[14] Larouche R, Saunders TJ, Edward G, Faulkner J, Colley R, Tremblay M (2014). Associations between active school transport and physical activity, body composition, and cardiovascular fitness: a systematic review of 68 studies. J Phys Act Health.

[15] Voss C, Winters M, Frazer A, McKay H (2015). School-travel by public transit: rethinking active transportation. Prev Med Reports.

[16] Buliung RN, Mitra R, Faulkner G (2009). Active school transportation in the greater Toronto area, Canada: an exploration of trends in space and time (1986-2006). Prev Med.

[17] Clark AF, Bent EA, Gilliland J (2016). Shortening the trip to school: examining how children’s active school travel is influenced by shortcuts. Environ Plan B Plan Des.

[18] Lu W, Lisako E, Mckyer J, Lee C, Goodson P, Ory MG (2014). Perceived barriers to children’s active commuting to school: a systematic review of empirical, methodological and theoretical evidence.

[19] McMillan TE (2005). Urban form and a Child’s trip to school: the current literature and a framework for future research. J Plan Lit.

[20] Loebach JE, Gilliland JA (2016). Free range Kids? Using GPS-derived activity spaces to examine Childrens neighborhood activity and mobility. Environ Behav.

[21] ParticipACTION (2016). The ParticipACTION Report Card on Physical Activity for Children and Youth ARE CANADIAN KIDS TOO TIRED TO MOVE?.

[22] Loebach J, Gilliland J (2016). Neighbourhood play on the endangered list: examining patterns in children’s local activity and mobility using GPS monitoring and qualitative GIS. Child Geogr.

[23] Glasgow RE, Permanente K (2012). Perceived barriers to self-management and preventive behaviors.

[24] Line T, Chatterjee K, Lyons G (2010). The travel behaviour intentions of young people in the context of climate change. J Transp Geogr.

[25] Neuwelt P, Kearns R (2006). Health benefits of walking school buses in Auckland.

[26] Fusco C, Moola F, Faulkner G, Buliung R, Richichi V. Toward an understanding of childrens perceptions of their transport geographies: (non)active school travel and visual representations of the built environment. J Transp Geogr. 2012;20:62–70.

[27] Holt NL, Spence JC, Sehn ZL, Cutumisu N (2008). Neighborhood and developmental differences in children’s perceptions of opportunities for play and physical activity. Health Place.

[28] Mitchell H, Kearns RA, Collins DCA (2007). Nuances of neighbourhood: Children’s perceptions of the space between home and school in Auckland. New Zealand Geoforum.

[29] Panter JR, Jones AP, van Sluijs EM, Griffin SJ (2010). Attitudes, social support and environmental perceptions as predictors of active commuting behaviour in school children. J Epidemiol Community Heal.

[30] Brunton G, Oliver S, Oliver K, Lorenc T (2006). A synthesis of research addressing children’s, young people’s and parents views of walking and cycling for transport. EPPI-Centre.

[31] Page AS, Cooper AR, Griew P, Jago R (2010). Independent mobility, perceptions of the built environment and children’s participation in play, active travel and structured exercise and sport: the PEACH project. Int J Behav Nutr Phys Act.

[32] Chillón P, Hales D, Vaughn A, Gizlice Z, Ni A, Ward DS (2014). A cross-sectional study of demographic, environmental and parental barriers to active school travel among children in the United States. Int J Behav Nutr Phys Act.

[33] Timperio A, Crawford D, Telford A, Salmon J (2004). Perceptions about the local neighbourhood and walking and cycling among children. Prev Med.

[34] Huertas-Delgado FJ, Herrador-Colmenero M, Villa-González E, Aranda-Balboa MJ, Cáceres MV, Mandic S, et al. Parental perceptions of barriers to active commuting to school in Spanish children and adolescents. Eur J Public Health. 2017;ckw249 Available from: https://academic.oup.com/eurpub/article-lookup/doi/10.1093/eurpub/ckw249 10.1093/eurpub/ckw249 28108594

[35] Faulkner GE, Richichi V, Buliung RN, Fusco C, Moola F. What’s “quickest and easiest?”: parental decision making about school trip mode. Int J Behav Nutr Phys Act. 2010;7. Available from: 10.1186/1479-5868-7-62. 10.1186/1479-5868-7-62 PMC2924842 20691063

[36] Larouche R (2015). Built environment features that promote cycling in school-aged children. Curr Obes Rep.

[37] Carlson JA, Sallis JF, Kerr J, Conway TL, Cain K, Frank LD, et al. Built environment characteristics and parent active transportation are associated with active travel to school in youth age 12–15. Br J Sports Med. 2014;48:1634-39. 10.1136/bjsports-2013-093101 PMC4447304 24659503

[38] Giles-Corti B, Donovan RJ (2002). The relative influence of individual, social and physical environment determinants of physical activity. Soc Sci Med.

[39] Timperio A, Ball K, Salmon J, Roberts R, Giles-Corti B, Simmons D (2006). Personal, family, social, and environmental correlates of active commuting to school. Am J Prev med.

[40] Eyler AA, Brownson RC, Bacak SJ, Housemann RA (2003). The epidemiology of walking for physical activity in the United States. Med Sci Sports Exerc.

[41] Sallis JF, Owen N, Fisher E, Glanz K, Rimer B, Viswanath K (2008). Ecological models of health behaviour. Heal Behav heal Educ theory, res Pract.

[42] Stokols D. Translating social ecological theory into guidelines for community health promotion. Am J Heal Promot. 1996;10:282–98. 10.4278/0890-1171-10.4.282 10159709

[43] Oliver M, Badland H, Mavoa S, Witten K, Kearns R, Ellaway A, et al. Environmental and socio-demographic associates of children’s active transport to school: a cross-sectional investigation from the URBAN study. Int J Behav Nutr Phys Act. 2014;11. Available from: 10.1186/1479-5868-11-70. 10.1186/1479-5868-11-70 PMC4080694 24888516

[44] Larsen K, Gilliland J, Hess P, Tucker P, Irwin J, He M (2009). The influence of the physical environment and sociodemographic characteristics on children’s mode of travel to and from school. Am J Public Health.

[45] Mitchell CAC, Clark AF, Gilliland JAJ (2016). Built environment influences of Children’s physical activity: examining differences by Neighbourhood size and sex. Int J Environ Res Public Health.

[46] Cerin E, Saelens BE, Sallis JF, Frank LD (2006). Neighborhood environment walkability scale: validity and development of a short form. Med Sci Sports Exerc.

[47] Saelens BE, Sallis JF, Black JB (2004). Neighbourhood-based differences in physical activity: Anenvironment scale evaluation. Am J Public Health.

[48] Kerr J, Rosenberg D, Sallis JF, Saelens BE, Frank LD, Conway TL (2006). Active commuting to school: associations with environment and parental concerns. Med Sci Sports Exerc.

[49] Frank LD, Sallis JF, Saelens BE, Leary L, Cain K, Conway TL (2010). The development of a walkability index: application to the neighborhood quality of life study. Br J Sports Med.

[50] Mcleroy KR, Bibeau D, Steckler A, Glanz K. An ecological perspective on health promotion programs. Health Educ Q. 1988;15:351-377. 10.1177/109019818801500401 3068205

[51] Statistics Canada (2011). National household survey.

[52] ESRI (2017). ArcGIS Desktop v10.4.

[53] Corp IBM (2016). IBM SPSS statistics for windows.

[54] Stata Statistical Software (2013). Stata Corp.

[55] Hosmer DW, Lemeshow S, Sturdivant RX (2013). Applied logistic regression.

[56] Hilbe JM (2011). Logistic Regression. Int Encycl Stat Sci.

[57] Field A (2009). Disovering statistics using SPSS.

[58] Alparone FR, Pacilli MG (2012). On children’s independent mobility: the interplay of demographic, environmental, and psychosocial factors. Child Geogr Routledge.

[59] Lorenc T, Brunton G, Oliver S, Oliver K, Oakley A (2008). Attitudes to walking and cycling among children, young people and parents: a systematic review. J Epidemiol Community Health BMJ Publishing Group Ltd.

[60] Mitra R, Faulkner GE, Buliung RN, Stone MR (2014). Do parental perceptions of the neighbourhood environment influence children’s independent mobility? Evidence from Toronto, Canada. Urban stud.

[61] Rodríguez A, Vogt CA (2009). Demographic, environmental, access, and attitude factors that influence walking to school by elementary school-aged children. J Sch Health Blackwell Publishing Inc.

[62] McDonald N (2012). Is there a gender gap in school travel? An examination of US children and adolescents. J Transp Geogr.

[63] Ziviani J, Scott J, Wadley D (2004). Walking to school: incidental physical activity in the daily occupations of Australian children. Occup Ther Int.

[64] Christian HE, Villanueva K, Klinker CD, Knuiman MW, Divitini M, Giles-Corti B (2016). The effect of siblings and family dog ownership on children’s independent mobility to neighbourhood destinations. Aust NZ J Public Health.

[65] De Nazelle A, Nieuwenhuijsen MJ, Antó JM, Brauer M, Briggs D, Braun-Fahrlander C (2011). Improving health through policies that promote active travel: a review of evidence to support integrated health impact assessment. Environ Int.

[66] Babey SH, Hastert TA, Huang W, Brown ER (2009). Sociodemographic, family, and environmental factors associated with active commuting to school among US adolescents. J public health policy. Palgrave Macmillan UK.

[67] Hohepa M, Scragg R, Schofield G, Kolt GS, Schaaf D (2007). Social support for youth physical activity: importance of siblings, parents, friends and school support across a segmented school day. Int J Behav Nutr Phys Act.

[68] McDonald N, Aalborg A (2017). Why parents drive children to school: implications for safe routes to school programs. J Am Plan Assoc.

[69] Shaw B, Watson B, Frauendienst B, Redecker A, Jones T. Children’s independent mobility: a comparative study in England and Germany (1971–2010). Policy Studies Institute. London; 2013.

[70] Clark AF (2012). Understanding determinants of active travel.

[71] Clark AF, Scott DM. Barriers to walking: an investigation of adults in Hamilton (Ontario, Canada). Int J Environ Res Public Health. 2016;13. Available from: 10.3390/ijerph13020179. 10.3390/ijerph13020179 PMC4772199 26840328

[72] Zhu X, Lee C (2009). Correlates of walking to school and implications for public policies: survey results from parents of elementary school children in Austin. Texas J Public Health Policy Palgrave Macmillan UK.

[73] Carver A, Salmon J, Campbell K, Baur L, Garnett S, Crawford D (2005). How do perceptions of local neighborhood relate to adolescents’ walking and cycling?. Am J Health Promot.

[74] Panter JR, Jones AP, van Sluijs EM (2008). Environmental determinants of active travel in youth: A review and framework for future research. Int J Behav Nutr Phys Act BioMed Central.

[75] Skaggs DL, Early SD, D’Ambra P, Tolo VT, Kay RM (2006). Back pain and backpacks in school children. J Pediatr Orthop.

[76] Sallis JF, Prochaska JJ, Taylor WC (2000). A review of correlates of physical activity of children and adolescents. Med Sci sports Exerc.

[77] Davison KK, Werder JL, Lawson CT (2008). Children’s active commuting to school: current knowledge and future directions. Prev Chronic Dis Cent Dis Cont Prev.

[78] Mah SK, Nettlefold L, Macdonald HM, Winters M, Race D, Voss C (2017). Does parental support influence children’s active school travel? Prev med reports. Elsevier.

[79] Rutten GM, Savelberg HH, Biddle SJ, Kremers SP (2013). Interrupting long periods of sitting: good STUFF. Int J Behav Nutr Phys Act.

[80] Sallis J, Owen N, Glanz B, Rimer K, Viswanath K (2015). Ecological models of health behavior. Heal Behav heal Educ.

[81] Mammen G, Stone MR, Faulkner G, Ramanathan S, Buliung R, O’Brien C (2014). Active school travel: an evaluation of the Canadian school travel planning intervention. Prev Med.

[82] Pabayo R, Gauvin L, Barnett TA (2011). Longitudinal changes in active transportation to School in Canadian Youth Aged 6 through 16 years. Pediatrics.

[83] Buttazzoni AN, Van Kesteren ES, Shah TI, Gilliland JA. Active School Travel Intervention Methodologies in North America: A Systematic Review. Am J Prev Med Elsevier Inc. 2018:1–10. Available from: 10.1016/j.amepre.2018.04.007 10.1016/j.amepre.2018.04.007 29776785

[84] Active and Safe Routes to School (2016). School Travel Planning.

[85] de Vries SI, Hopman-Rock M, Bakker I, Hirasing RA, van Mechelen W (2010). Built environmental correlates of walking and cycling in Dutch urban children: results from the SPACE study. Int J Environ Res Public Health.

[86] Wong BY-M, Faulkner G, Buliung R (2011). GIS measured environmental correlates of active school transport: a systematic review of 14 studies. Int J Behav Nutr Phys Act.

[87] Aguinis H, Edwards JR (2014). Methodological wishes for the next decade and how to make wishes come true. J Manag Stud.

[88] Rosenman R, Tennekoon V, Hill LG (2011). Measuring bias in self-reported data. Int J Behav Healthc Res.

[89] Healy MA, Gilliland JA (2012). Quantifying the magnitude of environmental exposure misclassification when using imprecise address proxies in public health research. Spat Spatiotemporal Epidemiol.

[90] Duncan EK, Scott Duncan J, Schofield G (2008). Pedometer-determined physical activity and active transport in girls. Int J Behav Nutr Phys Act BioMed Central.

